# Degree of Conversion and Microshear Bond Strength of New Composite to Postcured Composite Resin Surface Treated With Ethyl Acetate

**DOI:** 10.1155/ijod/3791468

**Published:** 2025-09-18

**Authors:** Tayebeh Rostamzadeh, Seyedeh Maryam Tavangar, Fatemeh Golsorkhtabar

**Affiliations:** ^1^Department of Restorative Dentistry, School of Dentistry, Guilan University of Medical Sciences, Rasht, Iran; ^2^Aesthetic and Restorative Resident, Department of Restorative Dentistry, School of Dentistry, Guilan University of Medical Sciences, Rasht, Iran

**Keywords:** composite resins, ethyl acetate, Fourier transform infrared spectroscopy, polymerization, shear strength

## Abstract

**Objectives:** This study aimed to investigate the impact of ethyl acetate (E) surface treatment on the degree of conversion (DC) of postcured composite, measured by the Fourier transform infrared spectroscopy (FTIR) method, and its microshear bond strength to the new composite.

**Materials and Methods:** A total of 42 composite discs were prepared in a plexiglass mold with a thickness of 2 mm. Both sides of the discs were postcured in the Labolight LV-ӀӀӀ machine for 5 min. The composite discs were then divided into three groups, each containing 14 discs (*n* = 14), based on the surface treatment method used. It is worth noting that the mentioned division was employed randomly to ensure an unbiased study. The control group received sandblast and silane treatment. The second group was treated with E, while the third group underwent treatment with both E and silane (ESi). Cylindrical composite blocks were bonded to the prepared discs in a tygon tube. All groups underwent 5000 thermal cycles. The microshear bond strength was measured using a universal testing machine, and DC before and after treatment with E was determined through FTIR testing. Additionally, samples were examined under a stereomicroscope with a 40x magnification to investigate the failure mode.

**Results:** The microshear bond strength of the control group was significantly higher than that of the other two groups; however, no significant difference was observed between the E and ESi groups (*p*-value = 0.18). In the control group, cohesive failure whereas in the other two groups, adhesive failure was the most frequent. The FTIR results showed that the application of E resulted in an 11% reduction in DC.

**Conclusions:** The surface treatment of postcured composite, utilizing both chemical and mechanical methods, still results in better bond strength. Although the use of E led to a higher bonding potential of the postcured composite, it did not improve bond strength compared to the control group.

## 1. Introduction

Today, the popularity of composite restorations has surged due to the advancements in adhesive dentistry, curing systems, and significant improvements in both mechanical and optical properties. However, a key concern with composite materials is polymerization shrinkage, which can range from 1.5% to 3%. Polymerization shrinkage can lead to microleakage, postoperative sensitivity, pulp inflammation, marginal discoloration, and secondary caries [1]. Indirect composites are commonly utilized in the construction of inlays, onlays, overlays, veneers, and other types of restorations. These materials offer several advantages, including a higher degree of conversion (DC), better proximal contact and anatomical form, longer term color stability, more precise edge integrity, improved physical properties, and greater wear resistance [2]. The lifespan and durability of indirect composites can be significantly increased through the use of polymerization devices that generate heat, light, pressure, or a combination of these elements [3]. On the other hand, increasing DC and reducing the number of unsaturated double bonds in indirect composites can complicate bonding. To achieve a strong and durable bond between the postcured composite and the new composite, proper surface preparation methods are essential for effective bonding to tooth structure or for repairs.

Various surface preparation methods have been introduced to increase chemical and mechanical adhesion. Chemical methods include the use of phosphoric acid, hydrofluoric acid, silane, primers, universal adhesives, and various solvents. Mechanical methods encompass air abrasion with aluminum oxide particles, laser treatments, and roughening with dental burs. According to two systematic reviews and meta-analyses, a combination of mechanical and chemical methods results in the highest bond strength [4–6].

Research has introduced numerous surface preparation techniques for indirect composites. In one study, air abrasion was found to produce the highest microtensile bond strength when compared with noncontact Er, Cr: YSGG laser treatments and silicon carbide [7].

Another study demonstrated that sandblasting with aluminum oxide particles + silane resulted in significantly higher microshear bond strength compared to sandblast + hydrofluoric acid + silane and sandblast + Monobond Etch & Prime [8]. Additionally, research has shown that both surface preparation methods and aging time significantly impact bond durability, underscoring the recommendation to utilize a universal bond, especially after aging [9]. Another study indicated that groups undergoing sandblasting exhibited the highest bond strength, except for the “sandblast + HF + silane” group. Moreover, the lowest bond strength was noted in the groups treated with hydrofluoric acid, ethanol, and H_2_O_2_ [10].

While numerous studies have focused on surface preparation methods for indirect composite restorations, a standardized approach has yet to be established. Various chemical solvents, including dichloromethane, chloroform, and ethyl acetate (E), have been investigated for their potential to enhance the bonding between old and new acrylic materials [11]. One study evaluated the shear bond strength of acrylic teeth with different surface treatments when bonded to a resin-based denture. The strongest bond strength was found in the group treated with a dichloromethane–monomer mixture, followed by E, the control group, and the cyanoacrylate group [12]. In another study, the transverse strength of repaired resin base dentures was investigated using two treatment methods: E for 120 s and dichloromethane for 5 s. Both methods produced similar bond strengths; however, because of the carcinogenic nature of dichloromethane, E is recommended as a safer alternative with reduced toxicity [13]. Considering that dental composites share a similar polymeric structure with acrylic resins, the main difference is that the acrylic matrix typically consists of polymethyl methacrylate (PMMA). In contrast, the composite matrix contains cross-linked dimethacrylate monomers, such as bisphenol glycidyl methacrylate (Bis-GMA) or urethane dimethacrylate (UDMA). In the study by Tavangar et al. [14], the effectiveness of chloroform on bonding old and new composites was examined, given its successes reported in acrylic denture repairs. Tavangar et al. [14] concluded that after surface roughening, phosphoric acid did not increase shear bond strength; however, chloroform was shown to significantly improve bond strength, nearly matching that of the control group (without any surface treatment) [14].

E (CH_3_COOC_2_H_5_) is an organic, volatile, nonpolymerizable solvent known for its reasonable cost, low toxicity, and acceptable aroma, which makes it a popular choice for denture applications [11]. This material is not classified by the International Agency for Research on Cancer (IARC), which adds to its safety profile for surface treatment. E is used to bond acrylic teeth with high DC to acrylic denture base or to bond soft liners in relining dentures. By swelling the surface layer of the polymer, E facilitates the penetration of polymerizing materials, contributing to the formation of a high-quality polymer network.

Given the lack of prior studies using E on resin composites, this study aims to explore its potential application by analogy with PMMA systems, given their partial chemical similarities. We aimed to investigate the effect of E surface treatment on the DC of postcured composite by the Fourier transform infrared spectroscopy (FTIR) method, as well as its microshear bond strength to a new composite. The first null hypothesis posited that the microshear bond strength of the postcured composite surface treated with E would differ from that of the control group. The second null hypothesis stated that the DC of the postcured composite would demonstrate differences before and after surface treatment with E.

## 2. Materials and Method

This in vitro study was conducted after receiving ethical approval from the Ethics Committee of Guilan University of Medical Sciences under the ethics code of IR.GUMS.REC.1403.340. To determine the appropriate sample size, we calculated the Δ bond strength by taking the difference between the highest and lowest mean values and dividing it by the mean standard deviation [14]. Then, referring to the sample size table of variance analysis [15], a sample size of 14 was determined for each of the three main groups. Forty-two composite discs were prepared using a plexiglass mold with a thickness of 2 mm and a diameter of 7 mm. The molds were filled with a 2 mm layer of Luna composite (SDI, Viv, Australia) in A2 universal color. To ensure maximum surface smoothness, the molds were placed between two glass slabs. Each composite layer underwent curing for 20 s with a light-curing device (Bluedent, LED Smart, Bulgaria) set at an intensity of 1000 cm/mW. Prior to curing, the light intensity was calibrated using a 100-DigiRate LM radiometer [14]. To ensure complete polymerization, both sides of the discs were postcured using a Labolight LV-ӀӀӀ machine (GC, Japan) for 5 min. The composite discs were then randomly divided into three groups (*n* = 14) according to the chosen surface treatment method.

The first group, designated as the control group, underwent a sandblasting procedure utilizing 50 µm aluminum oxide. This process was conducted vertically for 10 s, maintaining a distance of 2 cm from the substrate and applying a pressure of 2 bar. To remove any residual sandblasting particles, the samples were subsequently placed in an ultrasonic cleaning device (Biem Ultrasonic Makina San. Ltd., Istanbul, Turkey) filled with distilled water for 5 min. Following this, the samples were washed and dried for an additional 5 s. For surface treatment, silane (Ultradent, USA) was meticulously applied using a microbrush for 1 min and dried for 5 s.

In the second group (E), the bonding surface was treated with E, which was allowed to remain on the surface for 2 min then dried for 5 s.

The third group (ESi) involved the application of silane following the treatment with E, mirroring the procedures of the first group. Immediately after surface treatment across all groups, the porcelain bonding resin (Bisco Inc., Schaumburg, IL, USA) was applied to the bonding surfaces of all samples in accordance with the manufacturer's instructions [10, 12, 16]. Cylindrical composite blocks were made in tygon tube made of transparent polyethylene with height of 2 mm and diameter of 1 mm. The composite was carefully packed into these tubes using a condenser and cured for 20 s. Subsequently, the tubes were expertly cut with a scalpel blade, allowing for the samples to be separated without applying any pressure.

All groups were subjected to 5000 thermal cycles of 5–55°C with 15 s dwell time using a Nemo mechatronic thermocycler (Mashhad, Iran). These 5000 thermal cycles are equivalent to 6 months of in vivo functioning [17]. Microshear bond strength was measured using a universal testing machine (SANTAM-20, Iran, Tehran) employing the ring wire technique, with a wire thickness of 0.2 mm and a loading speed of 0.5 mm/min. The microshear bond strength was calculated on the MPa scale by dividing the maximum force at which failure occurs by the cross-sectional area of the sample. Subsequently, the samples were examined under a stereomicroscope (EchoLab, DEVCO s.r.l., Italy) to investigate the modes of failure.

In this study, we categorized failures into three types. Adhesive failure was considered when more than 80% of failures occurred in the adhesive area or at the adhesive–composite junction. Cohesive failure was considered when more than 80% of failures were in the composite, and finally the mixed failure refers to the combination of the two previous cases [8]. It is worth mentioning that all steps were conducted under standard environmental conditions, at room temperature and relative humidity. To investigate the resulting surface alterations, we prepared scanning electron microscope (SEM) images of two composite discs: one treated with sandblast and the other with E. These images provide clear visual evidence of the changes induced by the treatments. To specifically investigate the effect of E on the DC of the composite discs, we measured the DC of the bonding surface of two composite discs, one before and the other after treatment using FTIR testing. As shown in Equation (1), the percentage of unreacted C = C bonds for the composite was calculated by comparing the absorbance peak area of aliphatic C = C bonds (at a wavelength of 1638 cm^−1^) to that of an internal standard (aromatic C = C at 1608 cm^−1^) before and after curing of the specimen [18].

(1) $$\begin{array}{c} \%\text{DC}=\left(1-\frac{\left[\frac{{\text{abs(aliphatic:C }=\text{ C)}}_{1638{\text{ cm}}^{-1}}}{{\text{abs(aromatic:C}\ldots \text{C)}}_{1608{\text{ cm}}^{-1}}}\right]\text{cured}}{\left[\frac{{\text{abs(aliphatic:C }=\text{ C)}}_{1638{\text{ cm}}^{-1}}}{{\text{abs(aromatic:C}\ldots \text{C)}}_{1608{\text{ cm}}^{-1}}}\right]\text{uncured}}\right)\times 100, \end{array}$$

The mean values of microshear bond strength were analyzed using one-way analysis of variance (ANOVA). For pairwise comparisons, the Tukey honest significant difference (HSD) test was employed. To compare the failure modes in the three groups, the chi-square test was employed. Additionally, the Spearman correlation coefficient test was conducted to assess the relationship between bond strength and failure mode. All statistical analyses were carried out using SPSS version 16 software, with a significance level set at *α* = 0.05.

## 3. Results

The highest microshear bond strength was observed in the control group (14.37 ± 3.97 MPa), and the lowest was in the E group (8.30 ± 2.97 MPa). The mean and standard deviation of microshear bond strength of the three groups are shown in Table 1. Pairwise comparisons show a significant difference between the microshear bond strength of the control group and the E group, as well as between the control group and the ESi group (*p*-values, respectively, *p* ≤ 0.001 and 0.01). However, no statistically significant difference was found between the E group and the ESi group (*p*-value = 0.18).

**Figure 1:**
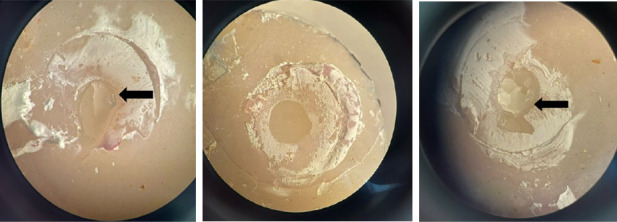
Stereomicroscope images from left to right include mixed failure in first group, adhesive failure in second group, and cohesive failure in second group, respectively. The black arrows indicate the remaining composite tube at the interface level. All images were captured at 40x magnification.

In the control group, the most frequent failure mode is cohesive failure, occurring 50% of the time. This is followed by mixed failure at 35.71% and adhesive failure at 14.28%. However, in the groups of E and ESi, adhesive failure has the highest frequency, occurring in 57.14% and 42.85% of cases, respectively. The mixed failure rates for these groups are 28.57% for E and 35.71% for ESi, while the cohesive failure rates are 14.28% for E and 21.42% for ESi. Images of the various failure modes, observed under a stereomicroscope, are displayed in Figure 1. The chi-square test revealed no statistically significant difference between the three study groups in terms of failure mode (*p*-value = 0.129).

Spearman's correlation coefficient indicates that there is no significant relationship between microshear bond strength and failure mode in the control group. In contrast, a significant relationship is observed in the E and ESi groups (*p* < 0.05).

**Figure 2:**
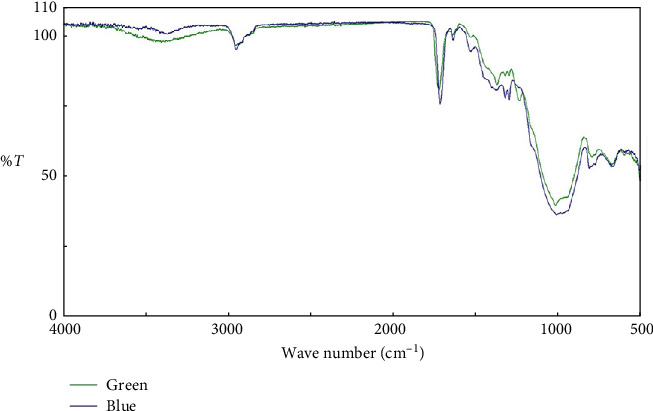
FTIR analysis of two postcured composite discs, one before applying ethyl acetate (blue) and the other after applying ethyl acetate (green).

Figure 2 illustrates the FTIR analysis of the two postcured composite discs: one before applying the E (blue) and one after (green). According to the DC formula, the DC of the postcured composite decreased from 75% to 64% after applying E.

**Figure 3:**
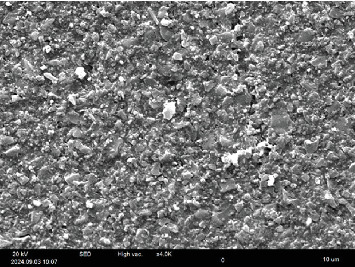
SEM image of composite disc treated with ethyl acetate surface treatment with 4000x magnification.

**Figure 4:**
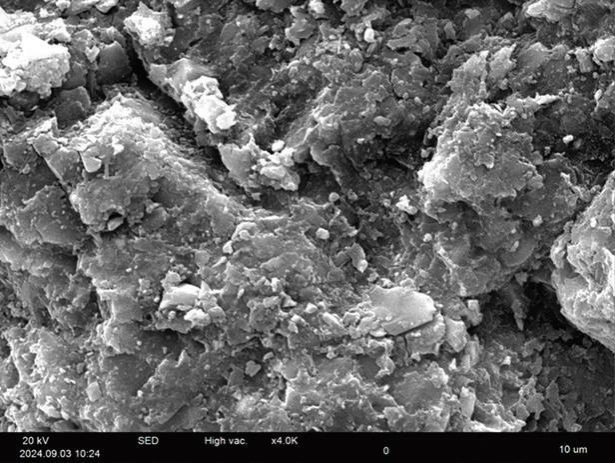
SEM image of composite disc treated with sandblast surface treatment with 4000x magnification.

Figures 3 and 4 present SEM images of two composite discs—one treated with E and the other subjected to sandblast. The images reveal that the disc treated with sandblast has a significantly rougher surface compared to the disc treated with E.

## 4. Discussion

In this study, we investigated the effect of E surface treatment on the DC of postcured composite using the FTIR method. We also examined its microshear bond strength to the new composite. The microshear bond strength test revealed that the bond strength of the control group was significantly higher than that of the other two groups (E and ESi). However, there was no significant difference between the two groups, E and ESi. This finding confirms the first null hypothesis, which refers to the difference in microshear bond strength between the E surface-treated postcured composite and the control group. Our findings highlight that still chemical and mechanical surface treatments together lead to better bond strength, as also observed in review articles [4–6]. The mean bond strength of the ESi group was higher than that of the E group, although this difference was not statistically significant. In a 2022 study, the bond durability of composite resin repaired with different surface treatments was investigated. The highest bond strength was observed in the group containing a diamond bur, phosphoric acid, and silane, which was significantly higher than in the other groups. Furthermore, the lower incidence of adhesive failure modes in groups containing silane in this study is in agreement with our findings, reinforcing the validity of our research, although this difference was not statistically significant in our study [19]. This result contradicts another study, which denoted that the use of silane, along with mechanical methods such as sandblast or diamond bur, did not improve the repair bond strength or durability of the bond. This study related the cause of this result to the chemical content of the used silane and the type of composite filler [20].

In the presented SEM images (Figures 3 and 4), a rougher surface with more porosities is seen in the sandblasted disc compared to the E-treated one. A study investigated the surface roughness of thermoplastic denture bases treated with E to enhance bonding with soft acrylic liners. Based on SEM images from the mentioned study, the surface roughness of the denture base was significantly increased after E treatment. The surface topography of these samples revealed surface dissolution and numerous pores, in contrast to the untreated control group. It has been stated that E facilitates the penetration of polymerizing materials and promotes the formation of a high-quality polymer network by swelling the surface layer of the polymer [21]. In various studies, E has been shown to be a safe chemical solvent for surface treatment of acrylic teeth with high DC to the denture base, as well as for relining the denture base with soft liner [11–13, 21, 22].

Postcuring converts the remaining free monomers into polymer and increases the DC. As the DC increases, the physical and mechanical properties of the resin materials improve [18]. However, the bonding potential of the resin may be diminished if repairs are necessary. According to the FTIR results, the DC of the postcured composite decreased by 11% after the application of E, and unsaturated carbon–carbon double bonds increased. As a result, in a postcured composite with a high DC, E leads to greater bonding potential. Therefore, the second null hypothesis, which pertains to the difference in DC of postcured composite before and after surface treatment with E, is confirmed.

One study investigated the repair strength of acrylic denture base surface treated with E at different times (5, 30, 60, and 120 s). The duration of 120 s had significantly higher bond strength than the other durations, but no significant difference was seen between durations of 5, 30, and 60 s [13]. As a result, in our study, a period of 120 s has been chosen for application of E. Although the application of E increased the bonding potential of the postcured composite, it did not improve the bond strength if compared to the control group. This could be attributed to the denser structure of composite resin in contrast to acrylics, as well as the limitations associated with in vitro studies. Therefore, further investigations, particularly in vivo studies are necessary to explore longer application times or multiple application protocols for E. It is worth noting that the excessive use of solvents can damage or weaken composites, and its effectiveness is also dependent on factors such as the type of composite used, its filler content, and the DC.

After the bond strength test, the failure mode of the samples was observed under a stereomicroscope. The control group primarily showed cohesive failure modes, whereas the other two groups demonstrated predominantly adhesive failures. However, the chi-square test revealed no statistically significant difference between the three study groups in terms of failure mode. According to Spearman's correlation coefficient, there was no significant relationship between microshear bond strength and failure modes in the control group. In contrast, a significant correlation was observed in the E and ESi groups, which showed a higher prevalence of adhesive failures. In a study, slight changes in the mean bond strength values for each material were observed depending on the type of their failure [2].

Future studies should consider testing E in combination with different composite types, varying filler content and monomer composition, to assess the generalizability of the current findings. The combination of mechanical surface treatments, such as sandblasting or diamond bur preparation, with chemical agents, such as E solvent and silane, as well as the potential for optimizing application protocols, such as concentration, exposure time, and multiple application protocol, should be explored to enhance clinical viability.

## 5. Conclusions

Based on the limitations of this in vitro study, we can draw the following conclusions:

1. The microshear bond strength in the control group was significantly higher compared to the other two groups (E and ESi). This suggests that a combination of mechanical and chemical surface treatments, as observed in the control group, still leads to higher bond strength.
2. The bond strength of the ESi group was found to be higher than that of the E group; however, this difference was not statistically significant.
3. The FTIR analysis revealed that after application of E, DC decreased by 11%. Consequently, using E on a postcured composite with a high DC can enhance bonding potential.

## Figures and Tables

**Table 1:** Pairwise comparisons of microshear bond strength (mean ± standard deviation).

(*I*) group	(*J*) group	*N*	Mean microshear bond strength (MPa)	Std. deviation	Minimum	Maximum	*p*-Value^a^
Control	E	14	14.37	3.97	7.57	19.78	≤0.001
	ESi	—	—	—	—	—	0.014

E	Control	14	8.30	2.97	4.68	14.74	≤0.001
	ESi	—	—	—	—	—	0.182

ESi	Control	14	10.60	3.07	7.02	16.28	0.014
	E	—	—	—	—	—	0.182

^a^The mean difference is statistically significant at the 0.05 level.

## Data Availability

The datasets analyzed during the current study are available from the corresponding author upon reasonable request.
